# Emerging evidence of endometrial compaction in predicting ART outcomes

**DOI:** 10.1093/hropen/hoae060

**Published:** 2024-10-10

**Authors:** Guangyao Lin, Stella Lim Jin Yie, Lianwei Xu

**Affiliations:** Department of Gynecology, Longhua Hospital, Shanghai University of Traditional Chinese Medicine, Shanghai, China; Department of Gynecology, Longhua Hospital, Shanghai University of Traditional Chinese Medicine, Shanghai, China; Department of Gynecology, Longhua Hospital, Shanghai University of Traditional Chinese Medicine, Shanghai, China

Sir,

We sincerely congratulate Al-Lamee *et al.* (2024) on their recent *Human Reproduction Open* article entitled ‘Endometrial compaction to predict pregnancy outcomes in patients undergoing assisted reproductive technologies: a systematic review and meta-analysis’. The authors provided valuable insights into the applicability of using endometrial compaction to predict ART outcomes by conducting a systematic review and meta-analysis of the available published data. Their results showed that endometrial compaction is significantly associated with favorable pregnancy outcomes, including clinical pregnancy rate (CPR) and ongoing pregnancy rate (OPR) compared to no endometrial compaction. However, in addition to the limitations reported by Al-Lamee *et al.* (2024), we have some concerns about the interpretation of the results obtained and the study methods used.

First, some important clinical variations were not considered by Al-Lamee *et al* (2024) when conducting the subgroup analyses: for example, factors contributing to infertility such as male factor, premature ovarian insufficiency, polycystic ovary syndrome, advanced age, etc. Additionally, different embryo quality and embryo stages may also serve as confounding factors when interpreting pregnancy outcomes. Therefore, to draw a more comprehensive understanding of endometrial compaction and its potential association with ART outcomes, we suggest that a more nuanced investigation of these variables should have been considered.

Second, publication bias may affect the reliability of this meta-analysis. We recommend the use of Egger’s test to objectively explore potential publication bias across the studies, thereby enhancing the validity of the pooled results (Higgins *et al.*, 2023). Further, although Al-Lamee *et al.* (2024) carried out sensitivity analyses to assess the robustness of their pooled results, a sensitivity analysis was only performed for the primary outcome, live birth rate (LBR). Hence, we would like to propose that a sensitivity analysis should also have been conducted for the secondary outcomes, including CPR, OPR, miscarriage rate (MR), positive pregnancy test, and rate of endometrial compaction, to convincingly determine the value of endometrial compaction in predicting ART outcomes.

Third, prospective cohort studies and retrospective cohort studies were included in the meta-analysis by Al-Lamee *et al.* (2024); however, this may lead to considerable heterogeneity owing to the different study designs. To investigate this heterogeneity, we suggest that if there are at least 10 studies in an analysis, Al-Lamee *et al.* (2024) could have adopted subgroup analyses or meta-regressions based on the different study designs to increase the credibility of their study (Higgins *et al.*, 2023).

Last but not least, three meta-analyses published prior to Al-Lamee *et al.* (2024) have also investigated the association between endometrial compaction and pregnancy outcomes. One study (Feng *et al.*, 2024) focused on the late-proliferative to secretory phase and concluded that endometrial compaction is not associated with ART outcomes, such as LBR, CPR, MR, OPR, biochemical pregnancy rate, and ectopic pregnancy rate, which was consistent with the results of the other two studies (Chen *et al.*, 2023; Turkgeldi *et al.*, 2023). However, in contrast, the meta-analysis by Al-Lamee *et al.* (2024) inferred that endometrial compaction is associated with CPR and OPR. Therefore, we suggest that the authors should have compared their results with other studies, which would have comprehensively strengthened the readers’ understanding of this field.
